# Expression of ASFV p17 in CHO cells and identification of one novel epitope using a monoclonal antibody

**DOI:** 10.1016/j.virusres.2023.199194

**Published:** 2023-08-23

**Authors:** Liwei Li, Sina Qiao, Shumao Wang, Jiachen Liu, Kuan Zhao, Yanjun Zhou, Guoxin Li, Yifeng Jiang, Changlong Liu, Guangzhi Tong, Wu Tong, Fei Gao

**Affiliations:** a Shanghai Veterinary Research Institute, Chinese Academy of Agricultural Sciences, Shanghai 200241, China; b College of Veterinary Medicine, Hebei Agricultural University, Baoding 071001, China; c College of Veterinary Medicine, Shandong Agricultural University, Tai'an, 271018, China; d Jiangsu Co-Innovation Center for the Prevention and Control of Important Animal Infectious Disease and Zoonose, Yangzhou University, Yangzhou 225009, China

**Keywords:** ASFV, P17, CHO cells, Monoclonal antibody, Epitope

## Abstract

As a highly pathogenic large DNA virus, African swine fever virus (ASFV) has huge particles and numerous encoded proteins. At present, few of the existing studies on ASFV proteins have investigated the function of p17. Specific antibodies against p17 to promote the development of prevention techniques against African swine fever (ASF) are urgently needed. Herein, we successfully expressed ASFV p17 in CHO cells using a suspension culture system and generated a monoclonal antibody (mAb) against p17. The mAb recognized a novel linear epitope (^8^LLSHNLSTREGIK^20^) and exhibited specific reactivity, which was conducive to the identification of ectopically expressed p17, the recombinant porcine reproductive and respiratory syndrome virus expressing p17, and the ASFV-SY18. The epitope was conservative among genotype I and genotype II ASFV strains. Overall, the mAb against p17 revealed efficient detection and promising application prospects, making it a useful tool for future vaccine research on ASF. Determination of the conserved linear epitope of p17 would contribute to the in-depth exploration of the biological function of ASFV antigen protein.

## Highlights

• ASFV p17 was successfully expressed in CHO cells using a suspension culture system.
• 1B4 recognized a conservative linear epitope (^8^LLSHNLSTREGIK^20^) among genotype i and genotype II ASFV strains.
• 1B4 effectively recognized the ectopically expressed p17 and foreign gene expression in recombinant PRRSV expressing ASFV p17.
• 1B4 can be a useful tool to evaluate the expression of p17 in the recombinant virus, making it meaningful for future vaccine research on ASF.

African swine fever (ASF) is an acute and highly contagious disease of domestic pigs and wild boars, which was first discovered in Kenya in 1921 (Dixon et al., 2019). There is no effective vaccine or drug available against this disease (Urbano and Ferreira, 2022; Wu et al., 2020). Since 2018, ASF virus (ASFV) outbreak occurred in China, and the emergence and prevalence of naturally occurring less virulent and naturally gene-deleted ASFV strains in domestic pigs have been identified in recent years (Gallardo et al., 2015, 2019; Sun et al., 2021a, 2021b; Zhao et al., 2019), leading to difficulties and challenges for control of ASF in China.

ASFV is the only member of Asfarviridae, and the only arbovirus DNA virus, which contains 170–193 kb DNA genome encoding more than 150 types of proteins (Dixon et al., 2013). Among these, the *D117L* gene encoding p17 is an abundant transmembrane protein localized at the viral internal envelope, which is essential for the progression of viral membrane precursors toward icosahedral intermediates (Suarez et al., 2010). p17 binds to the capsid protein p72 and closely connects the inner membrane and outer shell of ASFV virus (Wang et al., 2019). p17 can inhibit cGAS-STING signaling pathway to participate in complex interactions with the host for the benefit of the virus to evade the host's defenses (Zheng et al., 2022). In particular, p17 is detected as a specific antigen in the immunoreaction of pig sera with neutralizing antibodies (Munoz and Tabares, 2022). Given the above introduction, mAbs against ASFVp17 may serve as useful tools for viral research. It is important to identify exact epitopes before mAbs can be used for antigen detection, with pepscan analysis used frequently as a straightforward and reliable method (Cao et al., 2021). Herein, we expressed recombinant p17 of ASFV-18 strain in CHO cells using the suspension culture system, generate an anti-p17 mAb, which revealed efficient detection and promising application perspectives.

**Fig. 1:**
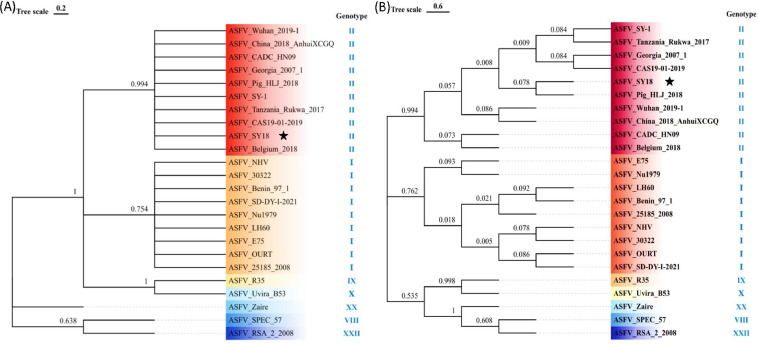
Phylogenetic trees were constructed based on the nucleotide sequences of *D117L* (A) and amino acid sequences of p17 (B). ASFV SY18 strain was marked with black asterisks. The scale bar indicated the number of nucleotide or amino acid substitutions per site.

First, we collected 24 representative ASFV strains of different genotypes from the GenBank database (Table 1) and multiple sequence alignments were performed using Clustal X. Phylogenetic trees of nucleotide sequences of *D117L* and amino acid sequences of p17 were constructed by MEGA 6.0 using the neighbor-joining method. Bootstrap confidence values from 1000 replicates were used to analyze the evolutionary relationship of SY18 p17. The results demonstrated that the nucleotide sequence and amino acid sequence of p17 from SY18 strain (marked with black star) both shared closer branches with genotype II strains (Fig. 1A-B).

**Table 1:** Reference strains in this study.

NO.	Isolate	Accession no.
1	ASFV Wuhan 2019–1	MN393476
2	ASFV China/2018/AnhuiXCGQ	MK128995
3	ASFV CADC_HN09	MZ614662
4	ASFV Georgia 2007/1	FR682468
5	ASFV Pig/HLJ/2018	MK333180
6	ASFV SY-1	OM161110
7	ASFV Tanzania/Rukwa/2017	LR813622
8	ASFV CAS19–01–2019	MN172368
9	ASFV-SY18	MH766894
10	ASFV Belgium 2018	LR536725
11	ASFV NHV	NC_044943
12	ASFV30322	MW736600
13	ASFV Benin 97/1	NC_044956
14	ASFV SD-DY-I-2021	MZ945537
15	ASFV Nu1979	MW723481
16	ASFV LH60	NC_044941
17	ASFV E75	NC_044958
18	ASFV OURT	NC_044957
19	ASFV 25185_2008	MW788410
20	ASFV R35	MH025920
21	ASFV Uvira B53	MT956648
22	ASFV Zaire	MN630494
23	ASFV SPEC_57	MN394630
24	ASFV RSA_2_2008	MN336500

**Fig. 2:**
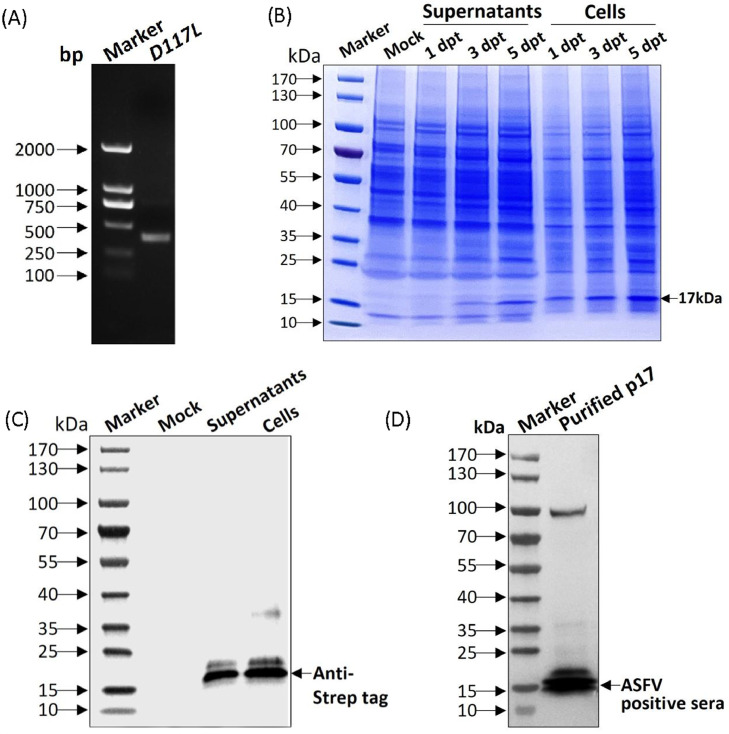
The recombinant p17 was successfully expressed and purified from CHO cells. (A) PCR amplification of *D117L* gene. (B) Identification of p17 expression in supernatants and cells at 1, 3, 5 days post-transfection by SDS-PAGE. (C) Identification of purified p17 by WB using anti-strep tag antibody. (D) Identification of purified p17 by WB using an ASFV-positive serum as primary antibody.

Next, we generated the pcDNA3.4-*D117L*-strep plasmid using the pcDNA3.4 TOPO™ TA Kit (Thermo Fisher Scientific, Waltham, MA, USA). ASFV *D117L* from SY18 (GenBank accession no. MH766894.1) was synthesized by Tsingke (Beijing, China). As shown in Fig. 2A, the *D117L* gene fragment was amplified by PCR amplification (Fig. 2A) and verified to be consistent with the reported gene sequence of ASFV SY18 strain by DNA sequencing. Sequences of all primers will be made available upon request. In recent years, the mammalian expression system is frequently and widely used (Fischer et al., 2015). The recombinant proteins obtained from this system have more effective and precise post-translational modifications. Serum-free and composition-simple medium for the suspension culture of mammalian cells contributes to massive purification and production and also increases the expression level of recombinant proteins. Moreover, viral envelope proteins, difficult to be expressed in prokaryotic systems, perform better in eukaryotic systems (Donaldson et al., 2022; Fischer et al., 2015). In this study, suspension-cultured CHO cells (Thermo) were transiently transfected by the eukaryotic expression vector pcDNA3.4-*D117L*-strep and maintained in a shaking incubator at 37 °C and 8% CO_2_ at 125r/min. Cells were incubated in a shaking incubator for 5 days and identified at 1, 3, and 5 days post-transfection (dpt). Cells were collected at 5 dpt, and the recombinant p17 was purified using StrepTrap beads according to the manufacturer's instructions (General Electric Company, Boston, MA, USA). The collected samples were identified by SDS-PAGE and Western blotting (WB) using an anti-strep tag antibody (1:4000, ab180957, Abcam, Cambridge, MA, USA). SDS-PAGE showed that the recombinant p17 was expressed successfully in both culture medium and ultrasonic supernatant of cells. The expression levels gradually increased with increasing transfection time and peaked at 5 dpt (Fig. 2B). We purified the recombinant p17 in the supernatants and cells at 5 dpt. Using an anti-strep tag antibody forming clear bands of approximately 17 kDa, the purified p17 was specifically detected, which was consistent with expectations (Fig. 2C). The purified p17 was identified by WB using ASFV-positive serum as primary antibody, and the purified protein was also specifically detected (Fig. 2D). Results above indicated that the recombinant p17 could be successfully obtained from suspension-cultured CHO cells and specifically recognized by ASFV-positive serum as a suitable antigen for subsequent tests.

The purified p17 was immunized into five four-week-old female BALB/c mice via intramuscular, subcutaneous, and intraperitoneal multipoint injections. All experimental programs involving mice were carried out in accordance with the Guidelines for the Nursing and Use of Experimental Animals, and approved by the Ethics Committee of Shanghai Veterinary Research Institute, Chinese Academy of Agricultural Sciences (SV-20210820–01). The animal experiments were guided by a professional veterinarian. The purified p17 (50 µg) was mixed with MnJ(β) colloidal manganese adjuvant. A second immunization was performed after 10 days, followed by a third and booster immunization in 7 days gaps. The spleen cells of mice and SP2/0 cells were hybridized to produce hybridoma cells. The purified p17 was coated onto enzyme linked immunosorbent assay (ELISA) plates at 200 ng/well, following incubation at 4 °C overnight. The ELISA plates were blocked with 5% skimmed milk in PBST at 37 °C for 1 h. The supernatants of hybridoma cells were added as primary antibodies at 37 °C for 1 h and HRP-conjugated goat anti-mouse IgG (*H* + *L*) (Proteintech Group, Inc., Chicago, USA) served as the secondary antibody following by incubation at 37 °C for 1 h, followed by TMB substrate (Beyotime Biotechnology, Shanghai, China) for 15 min. Hybridoma cells that secreted specific antibodies against p17 were screened using ELISA and subcloned four times. Monoclonal cells that stably secreted the specific antibody were finally selected to prepare the ascites. The ascites, named 1B4, were purified using a Pierce™ antibody clean-up kit (Thermo), and stored at −30 °C.

**Fig. 3:**
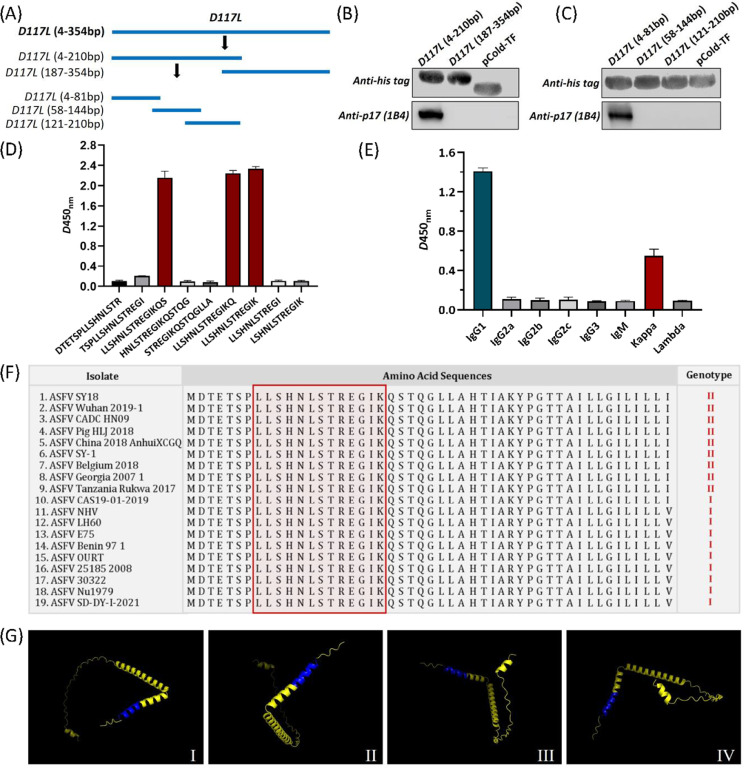
1B4 recognized specific linear B-cell epitope ^8^LLSHNLSTREGIK^20^. (A) Schematic diagram of *D117L*-truncated fragments. (B-C) A series of *D117L*-truncated fragments were constructed to pCold-TF and successfully expressed in *E. coli* BL21 (DE3) cells. 1B4 was used to detect the truncated p17 by WB using anti-His-tag antibody or 1B4 as primary antibody, respectively. (D) Nine different truncated peptides were synthesized and tested by ELISA to show the minimum epitope recognized by 1B4. (E) Identification of the mAb subtypes. The experiments included at least three independent repeats. Data are presented as the mean ± SD of three independent experiments. (F) Alignment analysis of the epitope (^8^LLSHNLSTREGIK^20^) among 19 representative ASFV strains from genotype I and genotype II using MEGA. (G) Prediction of the p17 structure using PyMOL. The epitope recognized by 1B4 is displayed in blue color.

As an important antigen protein of ASFV, our understanding about the epitopes of p17 is very limited. One recent study reports a specific linear B-Cell epitope (^3^TETSPLLSH^11^), which was conservative among 24 representative ASFV strains from different genotypes (Li et al., 2022). In this study, the minimal epitope recognized by the mAb (1B4) was identified by WB and ELISA. A series of truncated fragments of *D117L* gene were ligated into the prokaryotic expression vector pCold-TF (3365; TaKaRa, Dalian, China) and expressed in BL21 cells (TaKaRa) using IPTG (1 mM). The truncated protein recognized by the mAb was verified by WB. The *D117L* gene (4–354 bp) was first divided into two segments (P1:4–210 bp; P2:187–354 bp) and the expression plasmids were constructed (Fig. 3A). WB showed that 1B4 recognized the P1 region only (Fig. 3B). Further, the P1 fragment was truncated into three segments (P1–1: 4–81 bp; P1–2: 58–144 bp; P1–3: 121–210 bp). WB showed that 1B4 only recognized the P1–1 region (Fig. 3C), indicating that the epitope located within the P1–1 region. For further precise identification of the epitope, nine different truncated peptides were synthesized and used to coat the ELISA plates. The results showed that 1B4 recognized three of the nine truncated peptides and the minimum epitope located at amino acids 8–20 of p17, with the sequence ^8^LLSHNLSTREGIK^20^ (Fig. 3D). The subtypes of 1B4 were identified using a monoclonal antibody isotype identification kit (Proteintech) according to the manufacturer's instructions and the results showed that the heavy chain of 1B4 was IgG2b, whereas the light chain was a kappa chain (Fig. 3E).

There have been identified 24 ASFV genotypes based on the 3′-end sequence of *B646L* gene, which encodes the major capsid protein p72 (Muangkram et al., 2015). Georgia-07-like genotype II ASFV emerged in China and spread to other Asian countries since (Zhao et al., 2019). Until now, genotype II ASFV strains are main epidemic strains in China. Genotype I ASFV also emerged in domestic pigs and caused chronic infection in China (Sun et al., 2021a). We chose 19 representative genotype I and genotype II ASFV strains (Table 1) to analyze the conservation of the epitope recognized by 1B4. The amino acid sequences of p17 were also analyzed in spatial characteristics by DNASTAR software. Alignment of amino acid sequences showed that the epitope (^8^LLSHNLSTREGIK^20^) in 19 representative strains was 100% conserved (Fig. 3F). The prediction of the p17 structure by PyMOL revealed that the epitope (^8^LLSHNLSTREGIK^20^) was located at the N-terminal of p17 (blue) and exposed to the surface of the molecule (Fig. 3G). The prediction of p17 structure showed that the epitope was located at the N-terminus of p17 and was exposed to the surface of the molecule, contributing to the understanding of ASFV proteins. Results above indicated that ^8^LLSHNLSTREGIK^20^ was a conserved epitope of p17 among the representative ASFV strains from genotype I and genotype II.

**Fig. 4:**
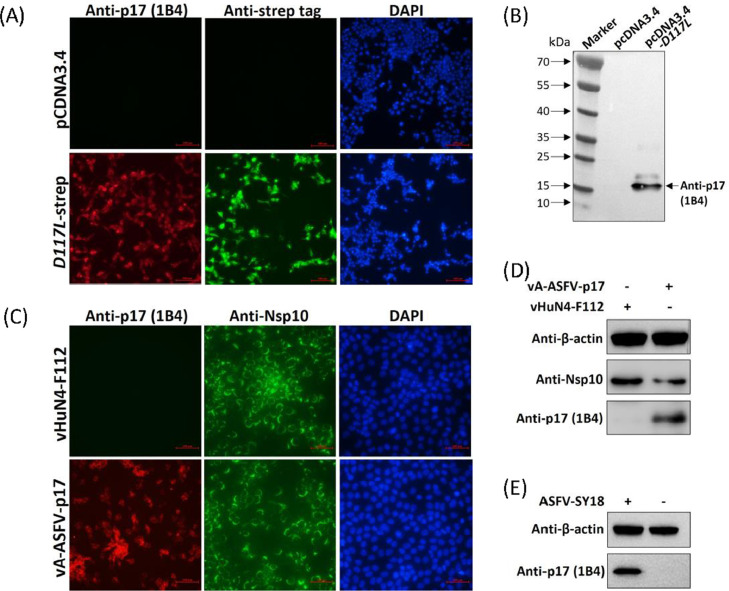
1B4 specifically recognized ectopically expressed p17 and recombinant PRRSV expressing p17. (A) 293T cells were transfected with pcDNA3.4-*D117L*-strep or control plasmid. Cells were fixed at 24 h post-transfection and immunostained with 1B4 as primary antibody and FITC-conjugated goat anti-mouse IgG as second antibody. Cellular nuclei were counterstained with 1 μg/ml of 4′,6′-diamidino-2-phenylindole (DAPI). (B) WB was conducted as treated in (A) to show the reactivity of 1B4. (C) MARC-145 cells were infected by vA-ASFV-p17 and vHuN4-F112. IFA against PRRSV Nsp10 or ASFV p17 in MARC-145 cells at 36 hpi with vA-ASFV-p17 and vHuN4-F112 (MOI = 0.1). Cellular nuclei were counterstained with DAPI. Scale bar = 100 µm. (D) WB was conducted as treated in (C) to identify the cell lysates using 1B4 and anti-Nsp10 antibody. (E) PAMs were infected with the ASFV-SY18 (MOI = 1) for 72 h. Cell lysates were then subjected to WB using the 1B4 as a primary antibody, with β-actin used as a loading control.

Finally, we analyzed the reactivity of 1B4. 293T cells were transfected by pCDNA3.4 and pCDNA3.4-*D117L*-strep plasmids for indirect immunofluorescence assay (IFA) and WB analysis as described previously (Cao et al., 2021), except that 1B4 or anti-strep tag antibody were used mentioned above to verify the specificity of 1B4. IFA showed that 1B4 and anti-strep tag antibody specifically recognized the pCDNA3.4-*D117L*-strep-transfected CHO cells with red and green fluorescence, respectively, whereas the control did not show any fluorescence (Fig. 4A). WB showed that 1B4 recognized specific and clear band with a molecular weight of approximately 17 kDa, whereas it did not react with the control (Fig.4B), indicating that 1B4 exhibited specific reactivity against ectopically expressed p17. For the innovation and development of ASFV live vector vaccine, we constructed a series of recombinant PRRSV expressing the major antigenic proteins of ASFV, including ASFV p30, p72, p17, p12, p54, pK205R etc. We had selected some recombinant viruses above to immunize piglets and did the immune efficacy against ASFV challenge. For each recombinant virus, the corresponding detection methods and antibodies are required. The mAb obtained in this study will have a broad application prospect in the development of novel ASF live vector vaccines. In the current study, we used the attenuated PRRSV strain vHuN4-F112 obtained by serial cell passage (GenBank accession no. EF635006) and recombinant PRRSV expressing ASFV p17 (vA-ASFV-p17) to test the reactivity of 1B4. MARC-145 cells were infected by vA-ASFV-p17 and vHuN4-F112 at a multiplicity of infection (MOI) of 0.1. IFA against PRRSV Nsp10 (a laboratory-preserved antibody against PRRSV nonstructural protein 10) or ASFV p17 was performed at 36 hpi. Cellular nuclei were counterstained with 1 μg/ml of DAPI. The fluorescence was visualized using an inverted fluorescence microscope (Olympus Corporation, Tokyo, Japan). Cell lysates collected at 36 hpi were used for WB analysis with 1B4 and anti-Nsp10 antibody as primary antibodies at the same time. The results showed that 1B4 specifically bound to vA-ASFV-p17-infected MARC-145 cells, producing red fluorescence, whereas it did not react with vHuN4-F112-infected MARC-145 cells, producing no fluorescence. As a control, the anti-Nsp10 antibody was used as the primary antibody and reacted with both viruses to produce a specific green fluorescence (Fig. 4C). WB showed that 1B4 specifically recognized the vA-ASFV-p17 producing a specific band and did not react with vHuN4-F112. As a control, the anti-Nsp10 antibody was used as the primary antibody, which reacted with both viruses to produce specific bands (Fig. 4D). Finally, we analyzed porcine alveolar macrophages (PAMs) after infection with the ASFV-SY18 (GenBank accession no. MH766894.3, MOI = 1) at 72 hpi. WB showed that 1B4 specifically recognized the ASFV-SY18 producing a specific band, approximately 17 kDa, and did not react with the control cells (Fig. 4E). These results indicated that 1B4 exhibited a specific response and could be used for the detection of ectopically expressed p17, the recombinant virus expressing ASFV p17, and the ASFV-SY18.

In conclusion, recombinant ASFV p17 was successfully expressed and purified from CHO suspension-cultured cells. The mAb 1B4 specifically recognized transiently expressed p17, as well as specifically recognized recombinant PRRSV-infected cells expressing ASFV p17 and the ASFV-SY18. The mAb recognized a novel linear epitope (^8^LLSHNLSTREGIK^20^) of p17, which was conservative among genotype I and genotype II ASFV. These results indicate that the anti-p17 mAb generated in this study has strong specificity and potential to be used in both basic and applied research on ASFV.

## Author statement

All authors have contributed to, seen and approved the final and submitted version. We have no conflicts of interest to disclose.

## Declaration of Competing Interest

The authors declare that they have no known competing financial interests or personal relationships that could have appeared to influence the work reported in this paper.

## Data Availability

Data will be made available on request.
